# Is HPV the Novel Target in Male Idiopathic Infertility? A Systematic Review of the Literature

**DOI:** 10.3389/fendo.2021.643539

**Published:** 2021-03-08

**Authors:** Francesco Muscianisi, Luca De Toni, Gloria Giorato, Andrea Carosso, Carlo Foresta, Andrea Garolla

**Affiliations:** ^1^ Unit of Andrology and Reproductive Medicine, Section of Endocrinology, Department of Medicine, Centre for Male Gamete Cryopreservation, University of Padova, Padova, Italy; ^2^ Unit of Gynecology and Obstetrics, Section of Physiopathology of Reproduction and IVF, Department of Surgical Sciences, S. Anna Hospital, University of Torino, Torino, Italy

**Keywords:** human papillomavirus, HPV seminal infection, male infertility, anti-sperm antibodies, assisted reproductive technique, HPV treatment

## Abstract

Infertility is an important health problem that affects up to 16% of couples worldwide. Male infertility is responsible for about 50% of the cases, and the various causes of male infertility may be classified in pre-testicular (for example hypothalamic diseases), testicular, and post-testicular (for example obstructive pathologies of seminal ducts) causes. Sexually transmitted infections (STI) are increasingly widely accepted by researchers and clinicians as etiological factors of male infertility. In particular, several recent reports have documented the presence of HPV in seminal fluid and observed that sperm infection can also be present in sexually active asymptomatic male and infertile patients. In this review, we aimed to perform a systematic review of the whole body of literature exploring the impact of HPV infection in natural and assisted fertility outcomes, from both an experimental and a clinical point of view. Starting from *in-vitro* studies in animals up to *in-vivo* studies in humans, we aimed to study and evaluate the weight of this infection as a possible cause of idiopathic infertility in males with any known cause of conception failure.

## Introduction

Human Papillomavirus (HPV) is the etiological agent of the most common sexually transmitted infection worldwide, with an estimated 6.2 million new cases annually (1). HPV comprises a group of small non-enveloped epitheliotropic viruses with a double-stranded circular DNA genome made-up of 8000 bp. Its virion has an icosaedral shape, of 55 nm diameter, constructed of 52 capsomeres, each containing five molecules of the major capsid protein L1 and a smaller number of the minor capsid protein L2 (2). HPV consists of more than 200 genotypes, adapted to particular epithelial tissues, such as anogenital skin and mucosa (3). According to the basis of oncogenic potential, HPV can be divided in two different groups: high-risk (HR-HPV) and low-risk (LR-HPV). The former ones, that include the well-known 16 and 18 types, have been classified as oncogenic to humans according the International Agency for Research on Cancer (4), and may cause neoplastic transformations in the following epithelial areas: cervix, vagina, vulva, anus, penis and oropharynx (5). The latter ones, such us 6 and 11 types, are responsible of benign diseases such as genital warts (6). HPV infections are primarily contracted by direct contact of the skin or mucoses with an infected lesion. Genital HPV infection is largely transmitted through sexual intercourse, mostly insertive intercourses, although non-penetrative types of contact, such us genital-genital, oral-genital and manual-genital are also possible routes of transmission (7). Concerning the epidemiology of HPV infection, an evident difference occurs in the prevalence of the infection between females and males: while in the formers the prevalence is high in the first years after the sex debut and thereafter it decreases, in males the prevalence remains high during the whole life (8). Despite HPV-related diseases have been historically almost exclusively studied in females, recently, a growing interest is developing towards HPV infection in males. Recently it has been definitely demonstrated that, in addition to the well-known external genital areas, HPV virions may also be detected inside the male reproductive tract. In particular, it has been detected in male accessory glands were it can represent a possible cause of MAGI (male accessory gland infection), a condition which may play an important role in the impairment of seminal fluid and thus of fertility (9, 10). Finally, it was found in semen, both in exfoliated cells and even bound to spermatozoa (1, 11). Since the first studies which focused about this topic, it was clear that HPV does not enter the sperm cells, differently from the infection of epithelial cells and the exact localization of HPV in sperm was clarified by some important *in vitro* studies. Through immunofluorescence techniques, some authors clarified both the mechanism of sperm-HPV binding and its exact localization (12, 13). They reported that HPV-L1 capsid protein is able to bind the glycosamino-glycan Syndecan-I on the sperm surface and located in the equatorial region of the head. On this basis, different authors investigated the possible role of HPV semen infection in male infertility and recent metanalyses showed that this condition can impair couples fertility through different mechanisms (11, 14–16).

## Aims of This Review

The first aim of this review was to summarize the implications of HPV semen infection on the following topics: i) effect on sperm parameters, ii) development of anti-sperm antibodies, iii) impact on both natural and assisted reproductive outcomes, such us pregnancy rate and miscarriage rate. We also discussed *in-vitro* studies reporting the possible role of HPV infected spermatozoa on blastocyst development and trophoblastic invasiveness. Moreover, we summarized the diagnostic and therapeutic strategies available in infertile couples aimed to improve the reproductive outcome.

## Data Sources and Methods

Literature analysis was performed on the electronic databases Medline, Embase, ScienceDirect and the Cochrane Library, considering the time interval from January 1995 to October 2020. Key terms included: “HPV semen infection,” “HPV male infection,” “HPV and male infertility,” “HPV and anti-sperm antibodies,” “HPV and sperm parameters,” “HPV and sperm DNA fragmentation,” “anti-sperm antibodies and fertility,” “HPV-infected spermatozoa and fertilization,” “HPV and fertility outcome,” “HPV and blastocyst,” and “HPV and trophoblast.” We considered randomized trials, observational and retrospective studies, original articles having as topic the relationship between HPV sperm infection and the following items: altered sperm parameters, anti-sperm antibodies (ASA), sperm apoptosis, sperm DNA alteration and infertility. In the included studies, all known causes of male infertility had been ruled out. We also included experimental *in vitro* studies focused on the effects of HPV infection on oocyte fertilization, blastocyst development, and trophoblastic cell invasiveness. Also, studies describing the adjuvant administration of the HPV quadrivalent vaccine Gardasil (Merck Serono S.p.A., Milan, Italy) as a possible strategy to promote HPV clearance from semen in infected males, were included. An accurate analysis of the references of the main works was successively performed. We considered data from eligible studies separately, according to different topics: “HPV and impairment of sperm parameters,” “HPV and development of anti-sperm antibodies,” “HPV and impairment of natural and assisted fertility outcome.” Manuscripts were selected according to the use of the following methods: semen parameters and the related alterations were defined according to WHO laboratory manual for examination and processing of human semen (17) and Hamilton Thorn motility analyzer (18); HPV-DNA detection in spermatozoa, whole semen and fertilized oocyte, was performed by the use of polymerase chain reaction (PCR) and fluorescence *in situ* hybridization (FISH) techniques. The evaluation of apoptotic events, related to HPV, in spermatozoa and embryo was detected through a terminal deoxyribonucleotidyl transferase-mediated dUTP nick-end labelling assay (TUNEL) test, DNA-Comet assay, DNA Disk chip assay, and Cell Death Detection ELISA assays or SCSA (flow cytometric technique). Sperm washing procedures relied on two-layer isolate colloid wash, test-yolk buffer procedures, swim-up procedure, modified swim-up with enzymatic treatment (Hyaluronidase) and discontinuous Ficoll gradients. Foresta et al. (13) used the hamster egg–human sperm penetration test (HEPT) to assess the ability of HPV-infected spermatozoa to fertilize and transfer viral genome into oocytes.

## Results

**Figure 1:**
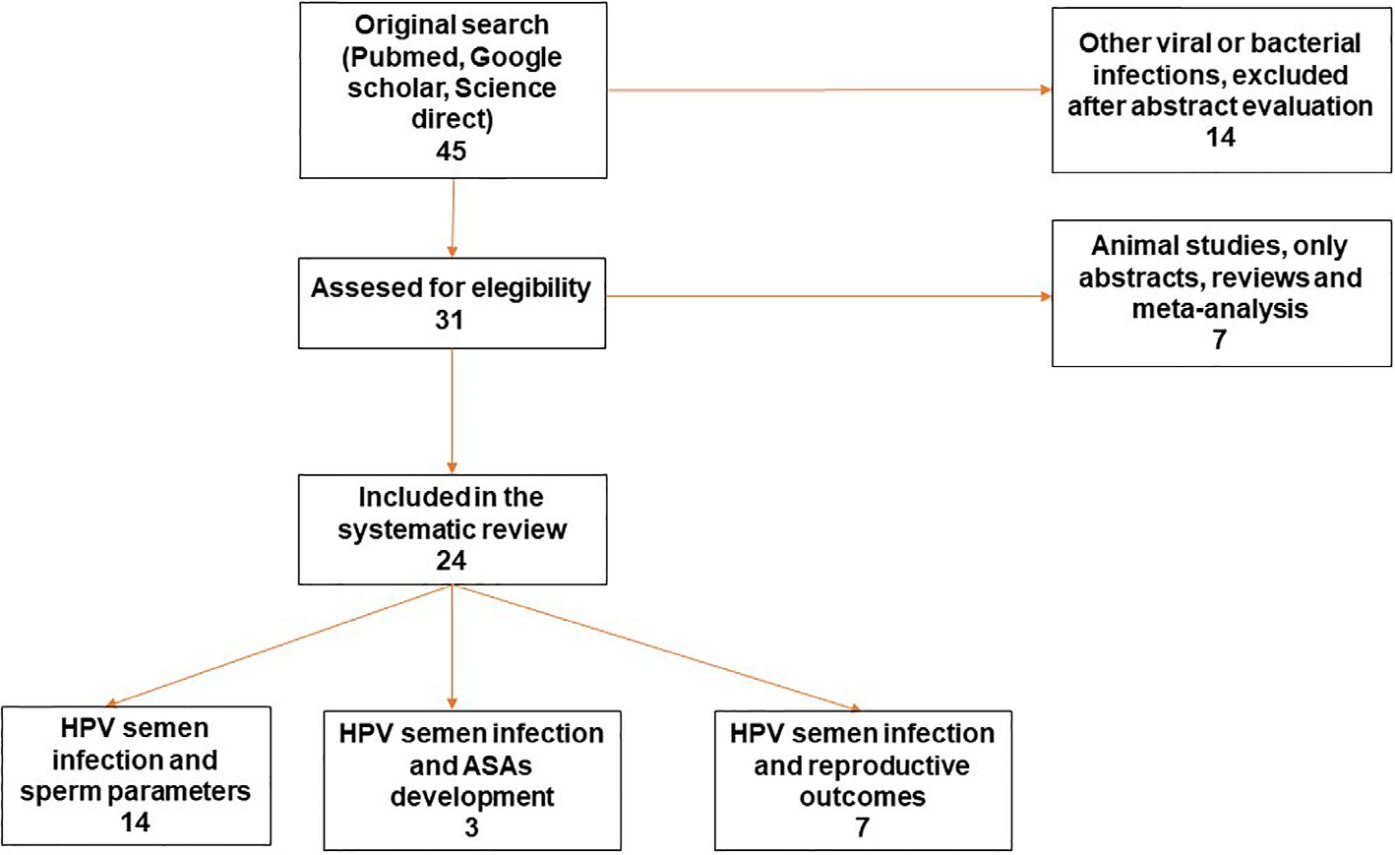
Preffered Reporting Items for Systematic Reviews and Meta Analyses (PRISMA) flow diagram of the review study on literature analysis for HPV semen infection and sperm parameters, ASAs development and reproductive outcome. Some of the studies evaluated more than a topic. HPV, Human Papillomavirus; ASAs, antisperm antibodies.

The literature search, based on previously mentioned key terms, identified a total of 24 papers meeting all the eligibility criteria for this review. **Figure 1** reports the Preferred Reporting Items for Systematic Reviews and Meta-Analyses **(**PRISMA) flow diagram which shows the process of study selection. The reported evidences ranged between 1997 and 2020. Some papers evaluated more than one topic. Among these manuscripts, 14 papers focused on the clinical impact of HPV detection in semen related to the alteration of sperm parameters (11, 14, 19–30); three papers (already included in the previous group) focused on the correlation between HPV semen infection in males, development of anti-sperm antibodies and their impact on male fertility (22, 25, 27) and seven papers (of which one already included in the first group) focused on the HPV semen infection in males and impairment of natural and assisted fertility outcomes (29, 31–36).

### HPV Semen Infection and Sperm Parameters

Because HPV infection is usually considered to be transient in males and without detrimental clinical consequences, its presence in semen has not been adequately investigated in the past. However, recently, an increasing number of studies have suggested the possible role of HPV in male infertility. In fact, several authors have confirmed the presence of the virus in the seminal fluid of men suffering from idiopathic infertility (15, 28, 37, 38). This data, combined with the higher prevalence of sperm HPV-infection in infertile subjects compared to general population (10%–35.7% *vs*. 2%–31%) (39) suggested that HPV may an important role in the impairment of sperm quality and, consequently, of male fertility.

**Table 1** reports the main studies which investigated the consequences of HPV seminal infections towards sperm quality seminal parameters and, consequently sperm quality. Among the 14 included studies reported, five reported a relation between HPV seminal infection and a reduced sperm motility (11, 14, 22, 24, 27). In addition, Piroozmand showed also a significant reduction of the sperm count. Boeri et al., documented an increased Sperm DNA fragmentation index when semen infections involved high-risk HPV genotypes (19) while Yang et al., reported that HPV seminal infection may cause an alteration of sperm normal morphology (30). In 2017, Damke et al. observed a correlation between HPV seminal infection and prostatic function, in terms reduced volume and increased pH and viscosity (20), while Rintala et al. demonstrated a seminal pH alteration in male patients with high-risk HPV seminal infection (28). Ultimately, Moghimi et al., observed a significantly higher prevalence of high-risk HPV in infertile men, compared to fertiles, associated with an impairment of sperm morphology and motility (26). Only four out of the 14 studies did not report any significant relationship between HPV seminal infection and sperm parameters (21, 23, 25, 29).

**Table 1:** Association between Human Papillomavirus (HPV) seminal infection and impairment of sperm parameters.

First Author	Country	Study setting	Kind of infertility	Years of infertility	Outcomes	Conclusions
**Boeri et al.** (19)	Italy	Cross-sectional s.	I. I.	N.A.	Volume, concentration, morphology, motility, leukocytes	HR-HPV genotypes induce reduced progressive sperm motility and increased sperm DNA fragmentation.
**Damke et al. (** 20 **)**	Brazil	Prospective cohort s.	I. I.	N.A.	Volume, concentration, morphology, motility, leukocytes	Altered prostate function with abnormal seminal volume, pH and viscosity.
**Fedder et al. (** 21 **)**	Denmark	Prospective cohort s.	N.O.A.	N.A.	Volume, concentration, sperm count	No impact on sperm parameters.
**Foresta et al. (** 11 **)**	Italy	Cross-sectional clinical s.	I. I.	N.A.	Volume, sperm count, concentration, motility, morphology	Reduced progressive sperm motility
**Foresta et al. (** 14 **)**	Italy	Cross-sectional clinical s.	N.A.	N.A.	Motility	Detrimental effect on sperm motility when HPV is bound to sperm
**Garolla et al. (** 22 **)**	Italy	Cross-sectional clinical s.	I. I.	2 years	HPV sperm infection, sperm aneuploidies, and sperm ASAs.	Reduced sperm motility and presence of ASAs, that may further reduce male fertility.
**Kim et al. (** 23 **)**	Korea	Cross-sectional s.	I. I.	N.A.	Volume, concentration, spem-count, motility, morphology	No impact on sperm parameters.
**Lai et al. (** 24 **)**	China	Cross-sectional clinical s.	I. I.	N.A.	Morphology, motility	Higher incidence of asthenozoospermia.
**Luttmer et al. (** 25 **)**	Netherlands	Cross-sectional s.	N. A.	N.A.	Volume, concentration, sperm-count, motility	No impact on sperm parameters
**Moghimi et al. (** 26 **)**	Iran	Case-control s.	I. I.	N.A.	Concentration, morphology, motility	HR-HPV genotypes induce reduced progressive sperm motility
**Piroozmand et al. (** 27 **)**	Iran	Cross-sectional s.	N. A.	N.A.	HPV sperm infection and presence of sperm ASAs	Reduced sperm count and motility.
**Rintala et al. (** 28 **)**	Finland	Cross-sectional s.	N. A.	N.A.	Volume, Sperm count, concentration, motility	Impairment of seminal pH.
**Tanaka et al. (** 29 **)**	Japan	Case-control s.	N. A.	N.A.	Concentration, motility	No impact on semen parameters.
**Yang et al. (** 30 **)**	China	Case-control s.	I. I.	N.A.	Volume, concentration, motility, morphology	Impaired sperm motility and morphology

HPV, Human papillomavirus; HR-HPV, High-risk Papilloma viruses; ASAs, Anti-sperm antibodies; s, study; I. I., Idiopathic infertility; N.O.A., Non-obstructive azoospermia; N.A., not applicable.

### HPV Semen Infection and Anti-Sperm Antibodies

In addition to the possible impairment of sperm parameters, different kind of semen infections have been associated with the development of anti-sperm antibodies (ASAs). Also HPV semen infection, has been recognized as a risk factor for the ASAs (40, 41). In fact, the prevalence of ASAs seems to be higher in infected infertile patients compared to non-infected infertiles and general population. Moreover, in infected infertile subjects, presence of antibodies is associated with a further reduction of sperm motility (42). Although the role of ASAs is controversial in reproduction, various mechanisms have been proposed as to how they affect male fertility, including sperm agglutination, impaired cervical mucus penetration, complement-mediated sperm injury through the female genital tract and interference with sperm-egg interaction (43). Different authors evaluated the role of HPV as an antigenic stimulus for the development of ASAs.

**Table 2** reports the main studies which investigated the relationship between HPV seminal infection, the development of ASAs and their implication in the worsening of fertility. In the study by Garolla et al., the authors studied the association between HPV infection and ASAs and their clearance time semen samples from infected and non-infected infertile subjects. They pointed out that more than 40% of HPV infected infertile patients had ASAs on the sperm surface. In contrast, this condition was significantly lower in non-infected infertile men and in fertile control subjects. Moreover, infected patients had a higher mean percentage of ASAs compared with non-infected ones. These findings suggested that sperm autoimmunity could probably be HPV-dependent. In order to confirm this finding, they documented the presence of both viral proteins and immunoglobulins in the same sperm cells of samples with positive sperm-mixed antiglobulin reaction (Mar) test results. Notably, when immunofluorescence for HPV 16-L1 was present on the sperm surface, they observed co-staining for IgA and IgG. This observation, according to the authors, suggested that semen infection could represent a new clinical condition associated with the presence of ASAs. Finally, they also reported that, in infected males, a significant viral clearance (approximately 85.3%) was obtained after 24 months of follow-up. Interestingly, the reduction in sperm infection paralleled the disappearance of ASAs and was significantly related to a progressive improvement of sperm motility (22). Likewise, Piroozmand et al. demonstrated that, compared with non-infected ones, patients with HPV semen infection had a higher rate of ASAs and a worse sperm quality, suggesting that young infertile couples should be tested for HPV and ASAs along with other causes of infertility (27). On the contrary, Luttmer et al. stated that the presence of HPV in semen was not associated with impaired semen parameters and presence of sperm ASAs (25).

**Table 2:** Association between Human Papillomavirus (HPV) seminal infection and presence of anti-sperm antibodies.

First Author	Country	Study setting	Kind of infertility	Years of infertility	Outcomes	Conclusions
**Garolla et al. (** 22 **)**	Italy	Cross-sectional clinical s.	I. I.	2 years	HPV sperm infection, sperm aneuploidies, and sperm ASAs.	Association with ASAs and long-lasting persistence
**Luttmer et al. (** 25 **)**	Netherlands	Cross-sectional s.	N. A.	N. A.	HPV sperm infection and presence of sperm ASAs	No association with ASAs.
**Piroozmand et al. (** 27 **)**	Iran	Cross-sectional s.	N.A.	N. A.	HPV sperm infection and presence of sperm ASAs	Association with ASAs.

HPV, Human papillomavirus; ASAs, Anti-sperm antibodies; s, study; I. I., Idiopathic infertility; N.A., not applicable.

### HPV Semen Infection and Reproductive Outcomes

Due to its ability to affect seminal parameters and to induce ASAs development, HPV semen infection seems to be a significant risk factor for male infertility. In this regard, many original studies, reviews and meta-analyses reported that HPV semen infection is related to reduced fertility both in natural and assisted conceptions. However, the exact mechanisms responsible for this condition have been never definitely assessed (16, 19, 31, 33, 39, 44, 45).

**Table 3** reports the main studies which investigated how HPV seminal infection in males may impair reproductive outcome in natural and/or assisted fertility. Depuydt et al., studying the HPV infection in semen samples (samples of sperm donors used for assisted reproductive techniques) coming from three different cryo-banks, observed that no pregnancies were obtained when using HPV-infected semen samples coming from sperm donors (31). The following year, in the context of the intra-uterine insemination technique (IUI), the detection of HPV virions in sperm from partners was associated with a negative IUI outcome (32). On this basis, they suggested to introduce in the counselling of infertile couples the examination of HPV in semen. In Italy, Garolla et al., reported that the presence of HPV in seminal fluid was associated with a reduction of both natural and assisted cumulative pregnancy rate and an increase in miscarriage rate (33). In another study, the same group reported that the administration of HPV quadrivalent vaccine Gardasil (Merck Serono S.p.A., Milan, Italy) as an adjuvant tool to counteract seminal infection was associated with enhanced clearance of HPV from semen and, in parallel, with an increased rate of natural pregnancies and live births in idiopathic infertile couples (34). Even Perino et al. observed a significant correlation between pregnancy rate and seminal HPV DNA in males of infertile couples compared to HPV- negative (66.7% *vs.* 15%) (35). On the contrary, neither Tanaka et al., nor Tangal et al. reported any significant correlation between HPV semen infection and adverse natural or assisted fertility outcomes (29, 36). However, their results can be explained as follows: the first study evaluated only HPV 16 infection, while all HPV types seems to have a role in infertility; the second, did not test the presence of HPV in the day of failed Intra-cytoplasmic sperm injection (ICSI) procedure, but only in a following moment.

**Table 3:** Association between Human Papillomavirus (HPV) seminal infection and natural and/or assisted fertility outcomes.

First Author	Country	Study setting	Kind of infertility	Years of infertility	Outcomes	Conclusions
**Depuydt et al. (** 31 **)**	Belgium	Prospective non interventional multicenter s.	N. A.	N. A.	Pregnancy rate in IUI from infected and non-infected sperm donors	No clinical pregnancy using infected sperm from donors
**Depuydt et al. (** 32 **)**	Belgium	Prospective non interventional multicenter s.	I. I.	N. A.	Pregnancy rate in IUI with infected and non-infected semen	Four times fewer clinical pregnancy using infected semen
**Garolla et al. (** 33 **)**	Italy	Cross-sectional clinical s.	I. I.	N. A.	Spontaneous and assisted reproductive outcomes (pregnancy rate, live births, and miscarriages) in infected and non-infected infertile couples	Reduction in natural and assisted cumulative pregnancy rate and increased miscarriage rate
**Garolla et al. (** 34 **)**	Italy	Case-control s.	I. I.	N. A.	Reproductive outcomes after HPV vaccination in infected infertile subjects	Better reproductive outcomes following HPV adjuvant vaccination
**Perino et al. (** 35 **)**	Italy	Prospective clinical s.	Oligo-astheno-teratozoospermia (58.6%) Idiopathic infertility (10.5%)	N. A.	Assisted reproductive outcomes in infected couples	Increased pregnancy loss using infected sperm
**Tanaka et al. (** 29 **)**	Japan	Case-control s.	N. A.	N. A.	In-vitro fertilization outcome in type 16 semen infection	No association with adverse fertility outcome
**Tangal et al. (** 36 **)**	Turkey	Prospective cohort s.	I. I.	N. A.	Prevalence of infection in failed ICSI cycles	No higher prevalence of HPV semen infection in patients with previous ICSI failures

HPV, Human papillomavirus; IUI, intra-uterine insemination; ICSI, intra-cytoplasmic sperm injection; s, study; I. I., Idiopathic infertility; N.A., not applicable.

### Effect of HPV Semen Infection on Embryo-Development (*In-Vitro* Studies)

Many *in-vitro* studies have shown a negative influence of HPV infection upon several aspects of human fertility. For example, Gomez et al., in 2008, reported that HPV transfected blastocyst and trophoblastic cells were affected by a reduction in decidua invasion capacity, potentially responsible for a failure of maternal uterine wall invasion by trophoblastic cell, subsequent placental dysfunction and adverse pregnancy outcome (46). Furthermore, several experimental studies have demonstrated the role of HPV in causing pregnancy loss by transmission of viral genes to oocytes and determining DNA fragmentation and apoptosis of embryonic cells (47, 48). However, there is little evidence regarding the possibility that HPV infected sperm are able to interfere with embryo development when injected into the oocyte cytoplasm (such as during the procedure of *in-vitro* fertilization). To better understand this process, Foresta et al. performed an *in-vitro* study evaluating the ability of the virus- infected sperm to transfer HPV DNA and capsid proteins to oocyte during fertilization. After transfecting a human sperm with a plasmidic episome containing HPV E6 and E7 proteins, they used the hamster egg-human sperm penetration test to show the ability of infected sperm to transfer the capsid protein L1 to oocyte and the expression of E6 and E7 viral protein in the fertilized oocyte (13). Their findings demonstrated that both spermatozoa transfected with E6 and E7 genes and exposed to HPV L1 capsid protein were able to penetrate the oocyte. These laboratory data, combined with the observation that HPV DNA is found in a larger proportion of abortions rather than voluntary termination of pregnancy (35), may suggest an active role for HPV (which is carried to the egg by the spermatozoa) in the etiology of premature term gestation. This phenomenon could lead to an increase in the fragmentation of embryonic DNA resulting in alteration and apoptosis of the embryo (13). It should be remembered, however, that the situations described above refer to *in vitro* models that may not reflect the *in vivo* situation, where the entry of viral DNA into the egg has never been proven (22). In fact, the actual state of literature is insufficient to draw definite conclusion regarding the effect of HPV infection on the most important reproductive outcomes following natural and assisted fertility in women.

## Possible Clinical Management of Infertile Infected Patients

It is well known that HPV infections frequently affect individuals for a long time and it has been suggested that certain anatomic sites could act as viral reservoirs able to sustain the persistence of the infection (39, 49). Nowadays, there is a lack of an effective and resolutive treatment for HPV infection and related problems, including the consequences of HPV infection on fertility. Therefore, it is mandatory, while waiting for the development of effective prevention and therapy solutions, to educate and counsel infected couples through the suggestion of strategies able to counteract the infection, especially when they are seeking fertility, both naturally and by assisted reproductive technologies (ARTs). Firstly, it is important to educate and provide careful counselling in couples where at least one of the members is infected with HPV. A 2014 controlled study showed the effectiveness of this strategy. Couples in which both partners had HPV infection at genital site, were carefully counselled to follow some strict advices aimed to clear the virus (like: hygiene of both of their reproductive tract and their hand; using personal underwear and personal towels only; avoiding oral and anal sex) and monitored at 6, 12, 18, and 24 months. Counselled couples had a significantly higher clearance rate and shorter time of viral persistence, compared to non-counselled infected controls (50). Furthermore, recent evidence has suggested that HPV vaccination is a valid tool even in patients who have already contracted the infection. In fact, it has been highlighted that patients with HPV semen infection receiving vaccination, had a faster rate of seroconversion and greater viral clearance, identified as the percentage of HPV DNA detectable in seminal fluid compared to infected patients who did not undergo vaccination (51). In the specific context of HPV and fertility outcome, a more recent study of 2018 analyzed 151 infertile couples in which the male partner had HPV semen infection. Among them, 79 males received the quadrivalent vaccination, while 71 refused it and served as controls. In 1 year of follow-up, an improvement of sperm parameters and natural fertility outcome was recorded in vaccinated patients compared to controls. In particular, vaccinated patients showed a higher viral clearance that paralleled an improvement of sperm motility and a reduction in the percentage of anti-sperm antibodies. Furthermore, couples where the male partner received vaccination recorded higher pregnancy and delivery rates and a lower miscarriage rate during the follow-up (34). In addition to counselling and adjuvant vaccination, different techniques of sperm selection (centrifugation, discontinuous density gradient and direct Swim-up) have been tested aimed to remove HPV from the sperm surface. However, all techniques had very poor or even absent effect in the complete removal of the virus. Very recently, our group tested a modified swim-up technique with the addiction of hyaluronidase enzyme obtaining the complete elimination of HPV from infected samples (52). The rationale of this treatment was to cleave the binding of HPV to its putative ligand, Syndecan-I located on the sperm surface (13). Compared to normal swim-up technique, the modified swim-up with hyaluronidase was able to abolish the binding between HPV and sperm in 100% cases of infected sperm, confirmed by negative fluorescent in-situ hybridization (FISH) for HPV, without any significant impairment of either motility or DNA fragmentation in the spermatozoa.

**Figure 2:**
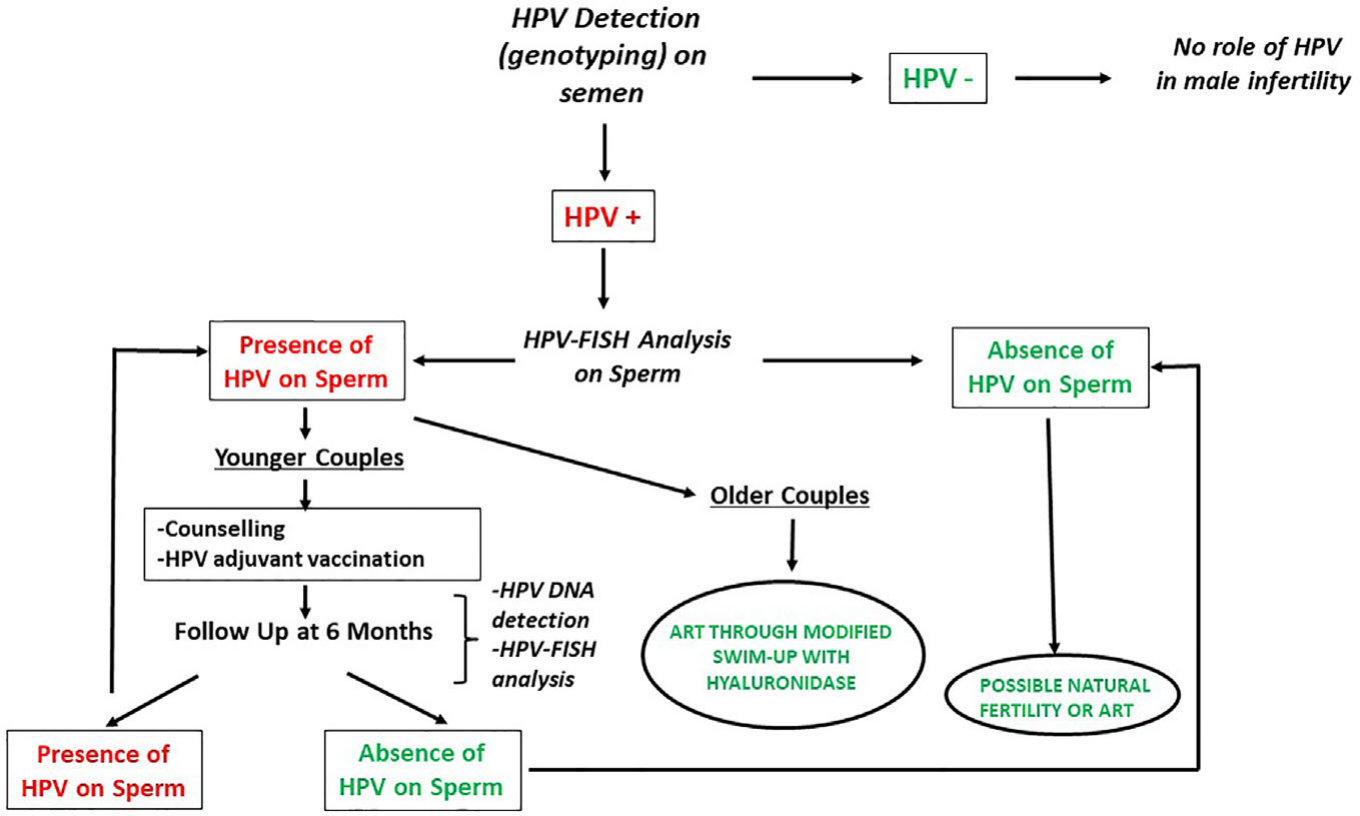
Management of infertile men candidate to HPV detection in semen (modified by Foresta et al. (39). Andrology). HPV, Human Papillomavirus; FISH, Fluorescent *in-situ* hybridization; ART, Assisted reproductive techniques.

On the basis of these evidences, we recommend testing for HPV in the male partner of the infertile couples in the following cases: male affected by unexplained couple infertility (not related to known male or female factors), asthenozoospermia, presence of ASA, positive medical history for HPV infection or evidence of ongoing HPV-related diseases. **Figure 2** describes the management suggested for infertile men candidate to HPV detection in semen. As a first line diagnosis, we suggest performing HPV detection and genotyping on semen by Polymerase-chain reaction (PCR) or by Inno-LiPA Genotyping Extra assay as previously described (22, 53). In negative cases, the role of HPV in male infertility can be excluded. In patients tested positive, a FISH analysis for HPV should be performed to detect the presence of HPV-DNA bound to sperm. In patients with a negative FISH analysis, it is possible to search for fertility both naturally or by ART. In patients showing the presence of HPV on the spermatozoa, the management is different in cases of younger or older couples since it should consider other clinical conditions and include a recommendation not to delay the initiation of infertility treatment. In younger couples, we suggest the previously described counselling, adjuvant vaccination and a new FISH analysis after 6 months of follow-up. If negative, couples may undergo through natural or assisted fertility seeking. If still positive, couples have to be redirected towards the same route or counselled to change strategy. In the latter case and in older couples we suggest performing ARTs through a modified technique of sperm selection by a modified swim-up with hyaluronidase to blunt virions attached to sperm.

## Conclusions

From the above reported literature, we can conclude that HPV semen infection can impair sperm quality and induce ASAs development, thus reducing couple fertility, particularly when viral DNA is present on the sperm surface. The impact of HPV genital tract infection in females is less clear, however the presence of the virus in embryos has been related to reduced pregnancy rate and increased abortion rate.

In the present systematic review, we summarize the state of the art on the link between HPV infection and couple infertility and suggest a flow-chart for the management and counselling of infertile couples in whom the male partner has HPV semen infection. The aim of this clinical management is to ensure the best reproductive outcome, both natural and through ARTs, particularly in those men without any other factor explaining infertility other than the presence of HPV. In the light of the large body of literature showing that HPV semen infection has a negative impact on reproductive outcome, we are strongly convinced that the time has come to follow this path. Further studies are required in order to demonstrate that the described strategy and, in particular, the clearance of HPV from semen is able to improve the delivery rate of infertile couples unable to conceive either naturally or *via* assisted reproduction.

## Strength and Limitations

The present review comprehensively summarizes, in a systematic way, the results of studies analyzing the correlation between HPV semen infection and male idiopathic infertility and provides the state of the art on the diagnostic and therapeutic strategies available in infertile couples aimed to improve the reproductive outcome. The main limitation of this analysis is that many of the studies, included in this review, did not consider important factors such as infertility duration and age of the female partner. Moreover, not all the studies clearly ruled out all known causes of infertility.

## Author Contributions

AG, FM, and CF designed the study. LT, FM, GG, and AC collected the data. LT and FM interpreted the results and drafted the report. All authors contributed to the article and approved the submitted version.

## Conflict of Interest

The authors declare that the research was conducted in the absence of any commercial or financial relationships that could be construed as a potential conflict of interest.

## References

[1] Dunne EF Nielson CM Stone KM Markowitz LE Giuliano AR. Prevalence of HPV infection among men: A systematic review of the literature. J Infect Dis (2006) 194:1044–57. 10.1086/507432 16991079

[2] Doorbar J Quint A Banks L Bravo IG Stoler M Broker TR. The biology and life-cycle of human papillomaviruses. Vaccine (2012) 30:F55–70. 10.1016/j.vaccine.2012.06.083 23199966

[3] Bouvard V Baan R Straif K Grosse Y Secretan B El Ghissassi F. WHO International Agency for Research on Cancer Monograph Working Group. A review of human carcinogens–Part B: biological agents. Lancet Oncol (2009) 10:321–2. 10.1016/S1470-2045(09)70096-8 19350698

[4] International Agency for Research on Cancer. Human papillomaviruses. IARC Monogr Eval Carcinog Risks to Hum (2007) 90:1–636. PMC4781057 18354839

[5] Cubie HA. Diseases associated with human papillomavirus infection. Virology (2013) 445:21–34. 10.1016/j.virol.2013.06.007 23932731

[6] de Sanjosé S Brotons M Pavón MA. The natural history of human papillomavirus infection Best Pract Res Clin Obstet Gynaecol (2018) 48:2–13. 10.1016/j.bpobgyn.2017.08.015 28964706

[7] Dunne EF Park IU. HPV and HPV-associated diseases. Infect Dis Clin North Am (2013) 27(4):765–78. 10.1016/j.idc.2013.09.001 24275269

[8] De Vuyst H Clifford G Li N Franceschi S. HPV infection in Europe. Eur J Cancer (2019) 45:2632–9. 10.1016/j.ejca.2009.07.019 19709878

[9] La Vignera S Vicari E Condorelli RA Franchina C Scalia G Morgia G. Prevalence of human papilloma virus infection in patients with male accessory gland infection. Reprod BioMed Online (2015) 30:385–91. 10.1016/j.rbmo.2014.12.016 25684094

[10] La Vignera S Condorelli RA Cannarella R Giacone F Mongioi’ L Scalia G. High rate of detection of ultrasound signs of prostatitis in patients with HPV-DNA persistence on semen: role of ultrasound in HPV-related male accessory gland infection. J Endocrinol Invest (2019) 42:1459–65. 10.1007/s40618-019-01069-8 31165424

[11] Foresta C Pizzol D Moretti A Barzon L Palù G Garolla A. Clinical and prognostic significance of human papillomavirus DNA in the sperm or exfoliated cells of infertile patients and subjects with risk factors. Fertil Steril (2010) 94:1723–7. 10.1016/j.fertnstert.2009.11.012 20056213

[12] Pérez-Andino J Buck CB Ribbeck K. Adsorption of human papillomavirus 16 to live human sperm. PloS One (2009) 4:e5847. 10.1371/journal.pone.0005847 19513123 PMC2689348

[13] Foresta C Patassini C Bertoldo A Menegazzo M Francavilla F Barzon L. Mechanism of Human Papillomavirus Binding to Human Spermatozoa and Fertilizing Ability of Infected Spermatozoa. PloS One (2011) 6:e15036. 10.1371/journal.pone.0015036 21408100 PMC3051064

[14] Foresta C Garolla A Zuccarello D Pizzol D Moretti A Barzon L. Human papillomavirus found in sperm head of young adult males affects the progressive motility. Fertil Steril (2010) 93:802–6. 10.1016/j.fertnstert.2008.10.050 19100537

[15] Laprise C Trottier H Monnier P Coutlée F Mayrand MH. Prevalence of human papillomaviruses in semen: a systematic review and meta-analysis. Hum Reprod (2014) 29:640–51. 10.1093/humrep/det453 24365799

[16] Lyu Z Feng X Li N Zhao W Wei L Chen Y. Human papillomavirus in semen and the risk for male infertility: a systematic review and meta-analysis. BMC Infect Dis (2017) 17:714. 10.1186/s12879-017-2812-z 29121862 PMC5679371

[17] World Health Organization. WHO Laboratory Manual for the Examination of Human Semen and Sperm-Cervical Mucus Interaction. World Health Organization, Cambridge, UK: Cambridge University Press (1999). Viral cancers.

[18] Burkman LJ. Discrimination between non hyperactivated and classical hyperactivated motility patterns in human sperm using computerized analysis. Fertil Steril (1991) 55:363–71. 10.1016/S0015-0282(16)54131-4 1991534

[19] Boeri L Capogrosso P Ventimiglia E Pederzoli F Cazzaniga W Chierigo F. High-risk human papillomavirus in semen is associated with poor sperm progressive motility and a high sperm DNA fragmentation index in infertile men. Hum Reprod (2019) 34:209–17. 10.1093/humrep/dey348 30517657

[20] Damke E Kurscheidt FA Balani VA Takeda KI Irie MMT Gimenes F. Male Partners of Infertile Couples with seminal infections of human papillomavirus have impaired fertility parameters. BioMed Res Int (2017) 2017:4684629. 10.1155/2017/4684629 28835893 PMC5556607

[21] Fedder J Ørnskov D Engvad B Kristensen TK Lomholt M Marcussen N. Seminal human papillomavirus originates from the body surface and is not a frequent aetiological factor in azoospermia. Andrologia (2019) 51:e:13202. 10.1111/and.13202 30565706 PMC7379244

[22] Garolla A Pizzol D Bertoldo A De Toni L Barzon L Foresta C. Association, prevalence, and clearance of human papillomavirus and antisperm antibodies in infected semen samples from infertile patients. Fertil Steril (2013) 99:125–31. 10.1016/j.fertnstert.2012.09.006 23043686

[23] Kim SJ Paik DJ Lee JS Lee HS Seo JT Jeong MS. Effects of infections with five sexually transmitted pathogens on sperm quality. Clin Exp Reprod Med (2017) 44:207–2013. 10.5653/cerm.2017.44.4.207 29376018 PMC5783918

[24] Lai YM Lee JF Huang HY Soong YK Yang FB. The effect of human papillomavirus infection on sperm cell motility. J Infect Dis (1997) 67:1152–5. 10.1016/S0015-0282(97)81454-9 9176459

[25] Luttmer R Dijkstra MG Snijders PJF Hompes PGA Pronk DTM Hubeek I Johannes Berkhof J. Presence of human papillomavirus in semen in relation to semen quality. Hum Reprod (2016) 31:380–6. 10.1093/humrep/dev317 26724799

[26] Moghimi M Zabihi-Mahmoodabadi S Kheirkhah-Vakilabad A Kargar Z. Significant Correlation between High-Risk HPV DNA in Semen and Impairment of Sperm Quality in Infertile Men. Int J Fertil Steril (2019) 12:306–9. 10.22074/ijfs.2019.5421 PMC6186290 30291691

[27] Piroozmand A Mousavi Nasab SD Erami M Hashemi SMA Khodabakhsh E Ahmadi N. Distribution of Human Papillomavirus and Antisperm Antibody in Semen and Its Association with Semen Parameters Among Infertile Men. J Reprod Infertil (2020) 21:183–8. PMC7362092 32685415

[28] Rintala MA Grénman SE Pöllänen PP Suominen JJ Syrjänen SM. Detection of high-risk HPV DNA in semen and its association with the quality of semen. Int J STD AIDS (2004) 15:740–3. 10.1258/0956462042395122 15537460

[29] Tanaka H Karube A Kodama H Fukuda J Tanaka T. Mass screening for human papillomavirus type 16 infection in infertile couples. J Reprod Med (2000) 45:907–11. 11127102

[30] Yang Y Jia CW Ma YM Zhou LY Wang SY. Correlation between HPV sperm infection and male infertility. Asian J Androl (2013) 15:529–32. 10.1038/aja.2013.36 PMC3739240 23603919

[31] Depuydt CE Donders GGG Verstraete L Vanden Broeck D Beert JFA Salembier G. Time has come to include Human Papillomavirus (HPV) testing in sperm donor banks. Facts Views Vis Obgyn (2018) 10:201–5. PMC6658204 31367292

[32] Depuydt CE Donders GGG Verstraete L Vanden Broeck D Beert JFA Salembier G. Infectious human papillomavirus virions in semen reduce clinical pregnancy rates in women undergoing intrauterine insemination. Fertil Steril (2019) 111:1135–44. 10.1016/j.fertnstert.2019.02.002 31005311

[33] Garolla A Engl B Pizzol D Ghezzi M Bertoldo A Bottacin A. Spontaneous fertility and in vitro fertilization outcome: New evidence of human papillomavirus sperm infection. Fertil Steril (2016) 105:65–72.e1. 10.1016/j.fertnstert.2015.09.018 26453270

[34] Garolla A De Toni L Bottacin A Valente U De Rocco Ponce M Di Nisio A. Human Papillomavirus Prophylactic Vaccination improves reproductive outcome in infertile patients with HPV semen infection: a retrospective study. Sci Rep (2018) 17:912. 10.1038/s41598-018-19369-z PMC5772512 29343824

[35] Perino A Giovannelli L Schillaci R Ruvolo G Fiorentino FP Alimondi P. Human papillomavirus infection in couples undergoing in vitro fertilization procedures: impact on reproductive outcomes. Fertil Steril (2011) 95:1845–8. 10.1016/j.fertnstert.2010.11.047 21167483

[36] Tangal S Taşçı Y Göksan Pabuçcu E Çağlar SG Haliloğlu AH Yararbaş K. DNA fragmentation index and human papilloma virus in males with previous assisted reproductive technology failures. Turk J Urol (2018) 45(1):12–6. 10.5152/tud.2018.96393 PMC6342580 29975635

[37] Chan PJ Su BC Kalugdan T Seraj IM Tredway DR King A. Human papillomavirus gene sequences in washed human sperm deoxyribonucleic acid. Fertil Steril (1994) 61:982–5. 10.1016/s0015-0282(16)56719-3 8174743

[38] Nielson CM Flores R Harris RB Abrahamsen M Papenfuss MR Dunne EF. Human papillomavirus prevalence and type distribution in male anogenital sites and semen. Cancer Epidemiol Biomarkers Prev (2007) 16:1107–14. 10.1158/1055-9965.EPI-06-0997 17548671

[39] Foresta C Noventa M De Toni L Gizzo S Garolla A. HPV-DNA sperm infection and infertility: From a systematic literature review to a possible clinical management proposal. Andrology (2015a) 3:163–73. 10.1111/andr.284 25270519

[40] Ochsendorf FR. Sexually transmitted infections: impact on male fertility. Andrology (2008) 40:72–5. 10.1111/j.1439-0272.2007.00825.x 18336453

[41] Rusz A Pilatz A Wagenlehner F Linn T Diemer T Schuppe HC. Influence of urogenital infections and inflammation on semen quality and male fertility. World J Urol (2012) 30:23–30. 10.1007/s00345-011-0726-8 21748371

[42] Heidereich A Bonfig R Wilbert DM Strohmaier WL Engelmann UH. Risk factors for antisperm antibodies in infertile men. Am J Reprod Immunol (1994) 31:69–76. 10.1111/j.1600-0897.1994.tb00849.x 8049027

[43] Francavilla F Santucci R Barbonetti A Francavilla S. Naturally-occurring antisperm antibodies in men: interference with fertility and clinical implications. An update. Front Biosci (2007) 12:2890–911. 10.2741/2280 17485267

[44] Siristatidis C Vaidakis D Sertedaki E Martins WP. Effect of human papilloma virus infection on in-vitro fertilization outcome: systematic review and meta-analysis. Ultrasound Obstet Gynecol (2018) 51:87–93. 10.1002/uog.17550 28608497

[45] Xiong YQ Chen YX Cheng MJ He WQ Chen Q. The risk of human papillomavirus infection for male fertility abnormality: a meta-analysis. Asian J Androl (2018) 20:493–7. 10.4103/aja.aja_77_17 PMC6116676 29623908

[46] Gomez LM Ma Y Ho C McGrath CM Nelson DB Parry S. “Placental infection with human papillomavirus is associated with spontaneous preterm delivery”. Hum Reprod (2008) 23(3):709–15. 10.1093/humrep/dem404 18184644

[47] Henneberg AA Patton WC Jacobson JD Chan PJ. Human papilloma virus DNA exposure and embryo survival is stage-specific. J Assist Reprod Genet (2006) 23:255–9. 10.1007/s10815-006-9030-8 PMC3506371 16871451

[48] Noventa M Andrisani A Gizzo S Nardelli GB Ambrosini G. Is it time to shift the attention on early stages embryo development to avoid inconclusive evidence on HPV-related infertility: debate and proposal. Reprod Biol Endocrinol (2014) 31:48. 10.1186/1477-7827-12-48 PMC4050410 24885125

[49] Giuliano AR Anic G Nyitray AG. Epidemiology and patholohy pf HPV disease in males. Gynecol Oncol (2010) 117:15–9. 10.1016/j.ygyno.2010.01.026 PMC4254924 20138345

[50] Garolla A Pizzol D Vasoin F Barzon L Bertoldo A Foresta C. Counseling reduces HPV persistence in coinfected couples. J Sex Med (2014) 11:127–35. 10.1111/jsm.12358 24165376

[51] Foresta C Garolla A Parisi S Ghezzi M Bertoldo A Di Nisio A. HPV Prophylactic Vaccination in Males Improves the Clearance of Semen Infection. EBioMedicine (2015) 2:1487–93. 10.1016/j.ebiom.2015.09.005 PMC4634690 26629543

[52] De Toni L Cosci I Carosso A Barzon L Engl B Foresta C. Hyaluronidase-based swim-up for semen selection in patients with human papillomavirus semen infection. Biol Reprod (2020) 10:ioaa173. 10.1093/biolre/ioaa173 33164043

[53] Foresta C Ferlin A Bertoldo A Patassini C Zuccarello D Garolla A. Human papilloma virus in the sperm cryobank: An emerging problem? Int J Androl (2010) 34:242–6. 10.1111/j.1365-2605.2010.01075.x 20522126

